# Real life data on nintedanib safety: idiopathic pulmonary fibrosis versus systemic sclerosis-interstitial lung disease and strategies adopted to manage adverse effects

**DOI:** 10.1007/s10787-023-01286-x

**Published:** 2023-08-03

**Authors:** Marco Di Battista, Laura Tavanti, Francesco Pistelli, Laura Carrozzi, Mattia Da Rio, Alessandra Rossi, Lorenzo Puccetti, Antonio Tavoni, Chiara Romei, Riccardo Morganti, Alessandra Della Rossa, Marta Mosca

**Affiliations:** 1 Rheumatology Unit, University of Pisa, via Roma 67, 56123 Pisa, Italy; 2 Department of Medical Biotechnologies, University of Siena, Siena, Italy; 3 Cardiovascular Thoracic Department, University of Pisa, Pisa, Italy; 4 Clinical Immunology and Allergy Unit, University of Pisa, Pisa, Italy; 5 Radiology Unit, University of Pisa, Pisa, Italy; 6 Section of Statistics, University of Pisa, Pisa, Italy

**Keywords:** Nintedanib, Systemic sclerosis, Idiopathic pulmonary fibrosis, Safety, Adverse effects, Management strategies

## Abstract

**Objective:**

Nintedanib (NIN) is an antifibrotic drug approved to slow the progression of idiopathic pulmonary fibrosis (IPF) and systemic sclerosis-related interstitial lung disease (SSc-ILD). NIN can frequently cause gastrointestinal adverse effects. We aimed to investigate the NIN safety profile in a real life setting, comparing IPF and SSc-ILD patients and evaluating the strategies adopted to manage NIN adverse effects.

**Methods:**

Patients taking NIN for IPF or SSc-ILD were enrolled. Alongside epidemiological and disease-specific data, the period of NIN use and the need for dosage reduction and/or interruption were investigated. Particular attention was paid to possible adverse effects and strategies adopted to manage them.

**Results:**

Twenty-seven SSc-ILD and 82 IPF patients were enrolled. No significant differences emerged between the two cohorts regarding the frequency of any possible adverse effect. Although the rates of NIN dosage reduction or interruption were similar between the two subgroups, SSc-ILD presented a mean period before NIN dosage reduction and NIN interruption significantly shorter than IPF (3 ± 2.6 vs 10.5 ± 8.9 months—p < 0.001 and 2.3 ± 0.5 vs 10.3 ± 9.9 months—p = 0.008, respectively). Several different strategies were tried to manage NIN adverse effects: especially in SSc-ILD, the variable combination of diet adjustment set by a nutritionist, probiotics and diosmectite was ultimately successful in maintaining patients on an adequate dose of NIN.

**Conclusion:**

We presented data on the NIN safety profile in a real life setting, which was similar between SSc-ILD and IPF. A combination of multiple managing strategies and dose adjustment appears essential to cope optimally with NIN adverse effects.

## Introduction

Nintedanib (NIN) is an intracellular inhibitor that targets multiple tyrosine kinases. This drug was first approved in 2014 for idiopathic pulmonary fibrosis (IPF) and then in 2019 for systemic sclerosis-related interstitial lung disease (SSc-ILD). NIN proved to be able to slow the progression of both diseases, reducing the decline in forced vital capacity (Distler et al. 2019; Richeldi et al. 2014). As emerged from several trials, NIN can be frequently associated with gastrointestinal adverse effects, thus negatively affecting its intake (Chen et al. 2021). The usual NIN starting dose is 150 mg bid, but when adverse effects occur it can be reduced to 100 mg bid or even suspended.

SSc is frequently burdened with gastrointestinal involvement, therefore a concomitant therapy with NIN could theoretically lead to a greater number of adverse effects: in this regard, consistent data from real life practice are scarce. The aim of this study was to investigate the NIN safety profile in a real life setting, comparing IPF and SSc-ILD patients and evaluating different strategies adopted to manage NIN adverse effects.

## Methods

Patients taking NIN for IPF or SSc-ILD in the tertiary referral centre of Pisa were retrospectively enrolled for this study. IPF data were collected from January 2016 to October 2022 in a pneumological setting, and SSc-ILD data collected from January 2019 to October 2022 in rheumatological and immunological settings. The local ethical committee approved this study (CEAVNO, n.854 prot.1199.262) and each patient voluntarily agreed to participate and gave written informed consent for the publication of the present study. The following data were prospectively collected from clinical charts:

- epidemiology and smoking habit;
- disease duration when starting NIN, need for oxygen therapy and deaths during the follow-up;
- months of NIN use and need to reduce to 100 mg bid and/or to suspend, evaluating after how long and for what main reason;
- adverse effects, distinguishing among diarrhoea, nausea, vomiting, liver-enzymes elevation, abdominal pain and bleeding;
- strategies adopted to manage adverse effects.

For SSc patients, disease-specific data were also collected regarding skin subset, autoantibody profile and concomitant intake of immunosuppressants.

### Statistical analysis

Continuous data were described by mean and standard deviation, categorical data by absolute and relative frequency. To compare the group variable with categorical and continuous factors, chi-square test and independent-samples t-test (two-tailed) were applied, respectively. To evaluate the influence of the group variable on the time to NIN dosage reduction or NIN interruption, a multiple linear regression was performed, indicating the regression coefficient (RC) with 95% confidence interval (CI). Significance was fixed at 0.05 and all analyses were carried out with SPSS v.28 technology (IBM Corp., Armonk, N.Y., USA).

## Results

Twenty-seven SSc-ILD (74% female, mean age 64.2 ± 11.6 years) and 82 IPF patients (18.3% female, mean age 74 ± 7.3 years) were enrolled for this study. Baseline characteristics of the cohorts, showing significant epidemiological differences due to the different nature of the diseases considered, can be found in Table 1. Most of the SSc patients had a diffuse cutaneous skin subset and anti-topoisomerase I positivity (74% for both), all of them previously took immunosuppressants and 16 (59.2%) were concomitantly taking an immunosuppressant together with NIN.

**Table 1:** Baseline characteristics of SSc-ILD and IPF cohorts

	SSc-ILD (n = 27)	IPF (n = 82)
Female (%)	20 (74)	15 (18.3)
Mean age ± SD years	64.2 ± 11.6	74 ± 7.3
Smoking habit (%) [former + active smokers, n]	7 (26) [6 + 1]	64 (78) [62 + 2]
Mean disease duration ± SD years	9.6 ± 8	2 ± 2
Oxygen therapy (%)	10 (37)	40 (48.7)

After a mean follow-up of 17.7 ± 7.4 months, 74% of SSc-ILD patients presented at least one adverse effect. NIN dosage reduction to 100 mg bid was necessary in 19 (70.3%) cases, whereas 3 (11.1%) patients had to suspend the drug. After a mean follow-up of 28.1 ± 19.1 months, 79.2% of IPF patients presented at least one adverse effect. NIN dosage reduction to 100 mg bid was necessary in 53 (64.6%) cases, whereas 15 (18.3%) patients had to suspend the drug. During the follow-up, death occurred in 4 SSc patients and in 24 IPF. A detailed characterization of the two NIN cohorts can be found in Table 2. In both subgroups, diarrhoea was the main adverse effect (66.6% vs 64.6%) and also the main reason for NIN dosage reduction (14 SSc-ILD cases—51.8% vs 36 IPF—43.9%) and NIN dose interruption (3 SSc-ILD cases—11.1% vs 9 IPF—10.9%).

**Table 2:** Comparison of NIN safety between SSc-ILD and IPF

	SSc-ILD (n = 27)	IPF (n = 82)	p
≥ 1 adverse effect (%)	20 (74)	65 (79.2)	0.572
Diarrhoea	18 (66.6)	53 (64.6)	0.848
Nausea	6 (22.2)	13 (15.8)	0.449
Vomiting	5 (18.5)	8 (9.7)	0.223
Liver-enzymes elevation	2 (7.4)	14 (17)	0.218
Abdominal pain	5 (18.5)	9 (10.9)	0.310
Bleeding	1 (3.7)	2 (2.4)	0.728
NIN dosage reduction (%)	19 (70.3)	53 (64.6)	0.585
Mean time to NIN dosage reduction ± SD, months	3 ± 2.6	10.5 ± 8.9	**< 0.001**
NIN dose interruption (%)	3 (11.1)	15 (18.3)	0.383
Mean time to NIN dose interruption ± SD, months	2.3 ± 0.5	10.3 ± 9.9	**0.008**
Deaths (%)	4 (14.8)	24 (29.2)	0.136

Significant *p*-values are shown in bold

When considering elderly patients (age ≥ 75 years, n = 49), no particular differences regarding adverse effects or NIN dosage reduction rate were reported. This subgroup presented instead a greater NIN dose interruption rate (24.4% vs 10%; p = 0.04).

Comparing SSc-ILD and IPF cohorts, no significant differences emerged regarding each adverse effect, the rate of NIN dosage reduction or NIN interruption and the rate of deaths (Table 2). The mean period before NIN dosage reduction to 100 mg twice daily was significantly shorter in SSc-ILD than IPF (3 ± 2.6 vs 10.5 ± 8.9 months; p < 0.001). Similarly, the mean period before NIN interruption was significantly shorter in SSc-ILD than IPF (2.3 ± 0.5 vs 10.3 ± 9.9 months; p = 0.008). A multivariate analysis was then performed taking into account sex, age, smoking habit, disease duration and follow-up duration. Time to NIN dosage reduction was reconfirmed significantly shorter in SSc-ILD patients even after multivariate analysis (RC -0.458; 95% CI -15.66/-2.99; p = 0.005), whereas time to NIN dose interruption was not reconfirmed (RC -0.177; 95% CI -23.67/13.36; p = 0.549).

When considering the SSc-ILD cohort, no significant differences emerged between sex, age, disease duration, smoking habit, skin subset, autoantibody profile or concomitant intake of immunosuppressants and any adverse effect, rate of NIN dosage reduction or NIN interruption. In particular, the concomitant intake of mycophenolate mofetil (11 patients) was not associated with an increased frequency of any gastrointestinal adverse effect.

Several different strategies were tried to manage NIN adverse effects, especially in SSc-ILD (Table 3). In fact, these patients usually required a variable combination of diet adjustment set by a nutritionist, probiotics and diosmectite. In IPF managing strategies were adopted less often and frequently consisted in a NIN dosage reduction with a total dose less than 100 mg twice daily (single dose 150 or 100 mg).

**Table 3:** Strategies to manage NIN adverse effects in SSc-ILD and IPF patients

	SSc-ILD (n = 20)	IPF (n = 65)
Strategy adopted (%)	13 (65)	9 (13.8)
Diet adjustment set by nutritionist	53.8%	22.2%
Diosmectite	46.1%	22.2%
Probiotics	38.4%	22.2%
Loperamide	15.3%	11.1%
Total dose less than 100 mg bid	15.3%	44.4%

## Discussion

Since its approval, NIN rapidly has become a pivotal drug in the therapeutic approach for IPF and SSc-ILD. However, the frequent association with adverse effects often limits its tolerability. So far, the NIN safety profile has been defined using data from some clinical trials (Cottin et al. 2022; Distler et al. 2019; Richeldi et al. 2014). The aim of this study was to investigate NIN-related safety concerns in a real life setting, highlighting potential differences between SSc-ILD and IPF. We also sought to evaluate which are the most common strategies adopted to manage adverse effects in these patients.

As expected, the two cohorts examined showed significant differences for almost all the epidemiological data. SSc-ILD patients in fact are more frequently female and slightly younger, whereas IPF subjects are usually older, smokers and predominantly male. Another confounding factor to consider is the different follow-up time of the cohorts, due to the different timing of drug approval. Despite those significant disparities, no significant differences emerged between SSc-ILD and IPF regarding any adverse effect or the rate of NIN dosage reduction or interruption. Our real life data did not show any new or cardiovascular NIN safety concern, and the rate of adverse effects was similar to that reported in a recent meta-analysis of 5 NIN trials (Chen et al. 2021). The only significant difference between SSc-ILD and IPF was the shorter time to NIN dosage reduction and interruption of the former. This finding, that in the case of NIN dosage reduction was not influenced by any confounding factor, has some possible explanations. First, SSc-ILD is frequently burdened with gastrointestinal involvement due to SSc itself. Therefore, if on the one hand SSc-ILD patients have the same frequency of adverse effects compared to IPF, on the other hand they could perhaps present a lower gastrointestinal tolerance threshold which leads to an earlier NIN dosage reduction or suspension. Another point to consider in SSc-ILD is the frequent concomitant intake of other drugs with gastrointestinal side effects. However, this argument seems less consistent given the absence of differences between SSc-ILD patients taking mycophenolate mofetil or not. Hence, our real life data strengthen the safety profile observed in the SENSCIS trial regarding the combination of NIN and mycophenolate mofetil (Highland et al. 2021).

When comparing our results with those of major clinical trials, we did not observe any gender difference in SSc-ILD patients regarding NIN safety concerns, whereas in the SENSCIS trial female SSc-ILD patients were reported to have more frequently nausea, vomiting, hepatic events and a higher rate of NIN dosage reduction and interruption (Seibold et al. 2020). However, the small number of our cohort, which is also the main limitation of the work, could have influenced this result. NIN proved to have a good safety profile in patients aged ≥ 75 years, although it was associated with a higher discontinuation rate in this subgroup, a fact already evidenced by various clinical studies (Glaspole et al. 2021).

Given the efficacy of NIN in reducing pulmonary progression, the need for a proactive management of adverse effects and dose adjustments is an essential element in clinical practice (Bendstrup et al. 2019; Glaspole et al. 2021; Seibold et al. 2020). Our data showed that SSc-ILD requires managing strategies more often than IPF. This could be due to the aforementioned higher gastrointestinal sensitivity of SSc patients. However, although multiple combined interventions are often required in SSc-ILD to achieve a similar rate of NIN dosage reduction or discontinuation compared to IPF, this same approach results in more frequent avoidance of excessive reduction (< 100 mg bid) of NIN posology, thus ensuring that patients receive an adequate dose of drug. Our results shows that in real life an optimal managing approach usually consists of the variable combination of diet adjustment set by a nutritionist, probiotics and diosmectite. This strategy, together with dose reduction to 100 mg bid, was effective in avoiding NIN discontinuation in the majority of patients who experienced adverse effects. Notably, although loperamide is recommended for the management of diarrhoea in IPF, its use in clinical practice is quite limited. This could be due both to the combination of other effective approaches and to the concerns regarding loperamide potential side effects in SSc patients (e.g. pseudo-obstruction).

## Conclusion

Although long-term data on NIN safety are already available from IPF trials (Crestani et al. 2019), our study brings the novelty of safety data from a real life setting, with a long-term follow-up of both IPF and SSc-ILD patients. The NIN safety profile in a real life setting is quite similar to SSc-ILD and IPF, and is comparable to what emerges from major clinical trials. A combination of multiple managing strategies and dose adjustment appears essential to cope optimally with NIN adverse effects.

## Data Availability

The datasets generated during and/or analysed during the current study are available from the corresponding author on reasonable request.
